# In search for the most optimal EEG method: A practical evaluation of a water-based electrode EEG system

**DOI:** 10.1177/23982128211053698

**Published:** 2021-10-26

**Authors:** Marta Topor, Bertram Opitz, Philip J. A. Dean

**Affiliations:** School of Psychology, University of Surrey, Guildford, UK

**Keywords:** Electroencephalography, cognitive neuroscience, event-related potential, time-frequency analysis, methodology, data quality

## Abstract

The study assessed a mobile electroencephalography system with water-based electrodes for its applicability in cognitive and behavioural neuroscience. It was compared to a standard gel-based wired system. Electroencephalography was recorded on two occasions (first with gel-based, then water-based system) as participants completed the flanker task. Technical and practical considerations for the application of the water-based system are reported based on participant and experimenter experiences. Empirical comparisons focused on electroencephalography data noise levels, frequency power across four bands (theta, alpha, low beta and high beta) and event-related components (P300 and ERN). The water-based system registered more noise compared to the gel-based system which resulted in increased loss of data during artefact rejection. Signal-to-noise ratio was significantly lower for the water-based system in the parietal channels which affected the observed parietal beta power. It also led to a shift in topography of the maximal P300 activity from parietal to frontal regions. The water-based system may be prone to slow drift noise which may affect the reliability and consistency of low-frequency band analyses. Practical considerations for the use of water-based electrode electroencephalography systems are provided.

## Introduction

Brain activity measured using electroencephalography (EEG) allows for a close investigation of electrical signals in the frequency and time domains. It is a common neuroscience method used in psychological, behavioural, cognitive and clinical research due to its affordability and ease of use. The standard state-of-the-art EEG equipment involves a swim-cap-like device with inserted electrodes. The connection between the scalp and the electrode is normally bridged with electrolyte gel. The signal is recorded through electrode wires connected to a computer. Technological advancements aim to improve the usability of EEG systems through new electrode types and wireless EEG signal recording.

The main disadvantage of the gold standard gel-based electrodes is the time-consuming preparation process including skin abrasion, gel application and impedance checks. The setup time depends on the number of included electrodes and researcher experience but typically varies between the average of 30 and 70 min (Kam et al., 2019; Oliveira et al., 2016). After the recording, participants have to wash the gel out of their hair and the electrodes require cleaning. Skin abrasion and lengthy preparation may be problematic for participants with sensory sensitivities, attention difficulties and restlessness which are often observed in young children and individuals with neurodevelopmental conditions such as autism spectrum disorder (ASD) or attention-deficit hyperactivity disorder (ADHD). The use of the systems is also limited to trained researchers or clinicians, but researchers often look for systems that can be applied independently by participants or patients at home (Hinrichs et al., 2020; Jochumsen et al., 2020; Radüntz, 2018).

Alternatively, systems that do not require skin abrasion such as EEG nets might be preferred (DiStefano et al., 2019; Pierce et al., 2021) though they generally use saline or gel solutions and cannot be used by participants independently. In the last few years, new systems with dry electrodes emerged. They require no skin abrasion, no gel/saline solutions and could potentially be used by participants independently without the presence of a trained researcher (Hinrichs et al., 2020; Kam et al., 2019; Pinegger et al., 2016). However, the data recorded with these systems have been reported to contain a higher number of artefacts (Hinrichs et al., 2020; Oliveira et al., 2016), higher pre-stimulus noise levels (Hinrichs et al., 2020; Mathewson et al., 2017) and lower signal-to-noise ratio (SNR; Radüntz, 2018) than standard gel-based electrode recordings. This is likely caused by the lack of an electrolyte substance that could bridge the scalp-electrode connection and keep the electrodes close to the skin throughout the recording (Mathewson et al., 2017; Pinegger et al., 2016). In addition, two studies reported lower participant comfort ratings for dry compared to wet EEG electrode systems due to the pressure from electrodes’ metal pins (Kam et al., 2019; Oliveira et al., 2016). These issues may deem dry-electrode systems unsuitable for many research designs.

Water-based electrodes are a promising development which could potentially improve on the gel-based systems’ disadvantages and mitigate the issues observed in dry-electrode recordings. They consist of plastic casings and paper or felt inserts soaked in tap water. Compared to gel-based electrodes, there is no need for skin preparation or washing hair and the preparation procedure is relatively easier and less time consuming. In contrast to dry electrodes, the scalp-electrode connection is supported with water which may help to sustain high-quality signal. No metal parts of the electrodes come into direct contact with the skin, thus potentially improving participant comfort as well. So far, the quality of EEG recordings using water-based electrodes has been evaluated in the context of brain computer interface (BCI) designs and the results are promising. Noise levels during a short circuit recording were the lowest in a water-based compared to gel-based and dry electrode systems (Pinegger et al., 2016) and the SNR has been reported to be comparable between water- and gel-based systems (Jochumsen et al., 2020). In addition, participant satisfaction was the highest for water-based compared to gel-based and dry systems (Pinegger et al., 2016). Moreover, the available water-based EEG systems allow for mobile wireless recordings of the EEG signals. This creates an opportunity to obtain EEG recordings in a wider range of contexts outside of the lab including everyday life situations, at home recordings, motor and sports research (Hinrichs et al., 2020; Oliveira et al., 2016; Radüntz, 2018). Taken together, water-based electrode EEG systems may seem very attractive for a wide range of research designs in neuroscience (examples of recent studies: Hazarika and Dasgupta, 2018; Raj et al., 2020).

To our knowledge, there are currently no empirical studies investigating the suitability of the new mobile water-based EEG electrode systems for application in cognitive and behavioural neuroscience research. We aimed to fill this gap by evaluating the quality of signal obtained with a water-based electrode system and investigating whether it may affect time-frequency and event-related potential (ERP) analyses. We also aimed to understand potential drawbacks and best methodological practices for the use of such systems. We applied evaluation methods and suggested benchmarking comparisons previously used for dry electrode systems evaluations (Hinrichs et al., 2020; Kam et al., 2019; Mathewson et al., 2017; Oliveira et al., 2016; Radüntz, 2018). Practical advice is provided alongside the obtained results for researchers who might want to consider using water-based electrode EEG systems in the future.

## Method

### Participants and procedure

The study consisted of two phases. Phase 1 was part of a procedure for an earlier study which used a gel-based EEG system (Topor et al., 2021b). The overall procedure during phase 1 was around 1 h and 45 min and included demographic and worry questionnaires (Penn State Worry Questionnaire; Meyer et al., 1990) and the Bruininks–Oseretsky Test of Motor Proficiency 2nd Edition–short form (BOT2-SF; Bruininks and Bruininks, 2005) prior to the EEG recording. Altogether, 46 participants were recruited in the pilot and the final study stage. Recruitment was facilitated through the University of Surrey’s research volunteer system and through word of mouth. All participants were given an opportunity to win one of two £50 prize vouchers and psychology students received lab tokens required as part of their course. Participants were excluded for diagnoses of psychiatric, neurological or neurodevelopmental disorders.

In phase 2, the same participants were contacted and invited to participate again. They were contacted one by one, in no particular order, and recruitment stopped when the 10th participant agreed to complete the study. Sample size was determined based on previous evaluations of EEG systems, which included eight to nine participants (Mathewson et al., 2017; Oliveira et al., 2016; Pinegger et al., 2016), and detected significant differences between the devices used. We also used a within-subject design which is advantageous for the preservation of power in studies with small sample sizes (Charness et al., 2012).

The final sample consisted of 10 participants who completed both phases 1 and 2. We attempted to ensure a gender balance within the sample and thus recruited five males and five females. Mean age at phase 1 was 26.5 years old, range of 22–38. The time between participation at phases 1 and 2 ranged from 7 to 12 months. In the final sample, there were no individuals who won the £50 prize in phase 1 and no additional incentives were offered at phase 2. Phase 2 consisted of the EEG recording and flanker task only and no additional demographic or health checks were carried out. All participants provided written informed consent. The study complied with ethical regulations at the University of Surrey, approval ID 428470-428461-48044088.

### Materials and equipment

#### Gel-based EEG system

The EEG recordings at phase 1 were acquired using a Brain Products setup with a gel-based EasyCap (EasyCap system kit, Brain Products, n.d.b; from now on referred to as the Brain Products setup) with 32 Ag/AgCl sintered electrodes in a 10/20 system (Fp1, Fp2, Fz, F3, F4, F7, F8, FC1, FC2, FC5, FC6, Cz, C3, C4, T7, T8, CP1, CP2, CP5, CP6, TP9, TP10, Pz, P3, P4, P7, P8, POz, O1 and O2). The ground electrode was located within the cap at the AFz position. The electrooculographic signal was recorded from the left side (vertical, VEOG) and above (horizontal, HEOG) the left eye using additional electrodes outside of the cap. The reference electrodes were also external to the cap, located on the mastoids and recorded implicitly (i.e. not as separate channels). Data were recorded in DC mode using Brain Vision Recorder V1.2 (Brain Products, 2012) at 500 Hz with amplifier input impedance at 10 GΩ and electrode impedance below 5 kΩ. A high cut-off online filter was implemented at 250 Hz.

**Figure 1.**
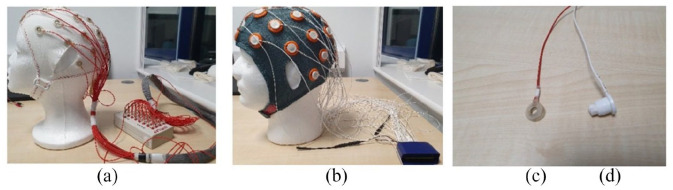
Two types of EEG recording setups and electrodes used in the current study: (a) Brain Products setup, (b) Mobita setup, (c) a gel-based Brain Products electrode and (d) a water-based Mobita electrode.

During the equipment preparation, each participant’s head circumference was measured with tape to select the right EEG cap size (52, 54, 56 or 58 cm available). The electrodes remained fitted within the caps between different recording sessions. Once the caps were placed on the head, the position of the electrodes was adjusted. The external electrooculographic and reference electrodes were placed on the skin using electrode stickers. Using a cotton bud, participant hair was moved from under the electrodes. We also applied an alcohol solution on the skin and the scalp directly through the hole in the electrodes (see Figure 1 for an illustration). This was followed by the application of the electrolyte gel directly at electrode locations. In the case of noisy channels, it was possible to improve the signal quality by reapplying the gel and securing electrodes closer to the scalp. The preparation of each participant for recording lasted from 30 min to 1 h.

The electrode cables were gathered at the back of the participant’s head in a tight net and plugged in an input box connected to Brainamp MR Plus amplifier (Brain Products, n.d.a). The EEG signal was recorded directly to a laptop that the amplifier was connected to using an USB adapter. The same USB adapter received the stimulus-response digital event markers from the stimulus computer via its parallel port. This wired setup enabled each stimulus and response category to have a unique signal at the parallel port translated to a unique marker value within Brain Vision Recorder alongside the EEG recording. All recordings were performed in a sound-proofed, electrically unshielded room.

#### Water-based EEG system

During phase 2, EEG data were acquired using the BIOPAC Mobita water-based setup (Mobita – W – 32 EEG, Biopac Systems, Inc., n.d.b; from now on referred to as Mobita) with 32 electrodes in a Mobita-32EEG-CAP-A ConfiCap (Biopac Systems, Inc., n.d.a). Similar to the Brain Products EasyCap cap, it had fixed electrode positions in the 10/20 system, though included Fpz and Oz channels instead of TP9 and TP10. In contrast to EasyCap, the ground electrode was not located within the cap but secured with a sticker in the middle of the forehead. It was not possible to add more external electrodes so the electrooculographic signal was extracted from Fp1 for VEOG and F7 for HEOG. The cap includes two reference electrodes located on the mastoids which are recorded as separate channels though the recording is performed in a reference-free mode. These mastoid electrodes can later be used for offline re-referencing. Recordings were obtained in DC mode at 1000 Hz using the Acqknowledge software V 5.0.3 (Biopac Systems, Inc., 2018). The Mobita system does not allow for the measurement of electrode impedance. It has been argued that electrode impedance may have little influence over data quality if amplifier input impedance is high (Ferree et al., 2001). However, Mobita’s input impedance was comparable to that of the Brain Products amplifier (10 GΩ) so we aimed to monitor possible noise interference for consistency between the two systems. Therefore, live spectral power was visually inspected for each electrode to detect noisy spikes at 50 Hz. Online filters were not applied.

Equipment setup included manual preparation of Mobita electrodes before participant arrival. Small pieces of absorbent paper (supplied by Biopac Systems, Inc.) were rolled and inserted into the plastic electrode casings. Electrodes were then placed in a jug of tap water. One adjustable cap size (medium: 54–58 cm) with empty holes (grommets) for the electrodes was fitted and adjusted for correct positioning for all participants. Although skin preparation is not suggested in the Mobita instruction manual (Biopac Systems, Inc., 2019), we decided to apply the same alcohol solution used in the Brain Products recording in the areas with exposed skin (forehead and mastoids) to remove the natural oiliness which could prevent good conductance for the water-based electrodes. However, alcohol makes the skin dry which could also reduce the skin to electrode connectivity in water-based systems (Biopac Systems, Inc., 2016), so we did not apply it anywhere else. Before inserting the electrodes, participant hair was moved with a cotton bud to expose the scalp within the empty grommets. If a noisy spike was observed at 50 Hz while checking the channels’ live spectral power, the electrode was removed, the hair was moved again to expose the scalp more and improve the electrode to scalp contact. In one case, due to noise across a number of electrodes, we tied a bandage around the participant’s head to keep the electrodes close to the scalp and prevent them from being dislocated by hair movement. Finally, the Mobita amplifier (Mobita-W-32EEG, Biopac Systems Inc., n.d.b) was placed in a sleeve and attached to participants’ right arm with a strap. The preparation procedure required 15 min prior to participant arrival and between 15 and 30 min in the presence of the participant (30–45 min in total).

The electrode cables were quite short, rested loosely at the participant’s back and were attached to the amplifier. The amplifier wirelessly transferred the EEG signal through a USB Wi-Fi antenna to a recording laptop. The stimulus computer was linked via its parallel port to the Digital I/O (37 pin) port of an STP100C module (isolated digital interface) attached to the MP160 Biopac device (Biopac Systems, Inc., n.d.c) which allowed for the digital event markers to be recorded using Acqknowledge. However, the EEG data stream from the Mobita amplifier and the event marker data stream from MP160 could not be integrated into one recording pane or synchronised across two recording panes in Acqknowledge (version 5.0.3). To solve this, a bespoke setup was made, whereby the event markers were sent via a wired connection to the Mobita amplifier to integrate into the recording at source. The integrated (EEG & event marker) data were then transferred wirelessly to the recording laptop as described before. Further details on this setup can be found in the Supplementary File. Recordings were performed in an electrically unshielded room with no special sound proofing as the system has been designed to be mobile and suitable for use in a wide range of environments. Table 1 displays a summary of technical differences between the two systems.

**Table 1.** A summary of technical differences between the Brain Products and Mobita EEG systems.

	Brain Products	Mobita
Non-overlapping channels	TP9, TP10	FPz, Oz
Ground electrode location	AFz	Middle of the forehead
Reference recording mode	Linked Mastoids (Implicit)	Reference Free Recording
Electrooculographic electrodes	Separate HEOG and VEOG	Fp1, F7
Electrode impedance	<5 kΩ	Not available
Online filter	250 Hz	None
Sampling rate	500 Hz	1000 Hz
EEG Cap size	Based on the head size	One adjustable size
Total preparation time	30–60 min	30–45 min
Recording room	Unshielded, sound-proofed	Unshielded

HEOG: horizontal electrooculogram; VEOG: vertical electrooculogram; EEG: electroencephalogram.

#### Cognitive task

The participants completed an arrow version of the flanker task (Eriksen and Eriksen, 1974) while the EEG data was acquired. This is a commonly used task in the study of attentional and error-control processes suitable for ERP research investigating both stimulus- and response-locked components such as P300 and error-related negativity (ERN; Pratt et al., 2011; Rietdijk et al., 2014). The task was presented using E-Prime software version 3 (Psychology Software Tools, 2012). Each trial consisted of seven arrowheads presented at the centre of the screen. The target stimulus was the middle arrowhead and participant’s task was to detect whether it was pointing left or right and respond using the computer keyboard (letter ‘C’ for left and letter ‘M’ for right). Three distractor arrowheads on each side of the target changed direction depending on the trial condition. If they pointed in the same direction as the target, the condition was congruent. If in the opposite direction, it was incongruent. In the neutral condition, the distractor arrowheads were replaced with the letter ‘v’. Each trial was proceeded by a fixation cross. Maximum response time in each trial was 600 ms and between-trial intervals were jittered in duration (400–1600 ms). One participant who initially participated in the study during the pilot completed 750 trials and all remaining participants completed 600 trials (200 per condition) with the task taking approximately 20 min.

#### Researcher and participant experience

Participants’ experiences of both systems were discussed at phase 2. Their observations were noted retrospectively and remain anecdotal in nature. However, they provide important practical information which should be considered for research protocols involving the Mobita system. Researcher notes focused on technical issues observed during the recordings. All observation notes can be found in the Demographics file in the project repository (https://osf.io/kubv5/; Topor et al., 2021a).

### Data analyses

#### Data import and digital marker positions

Offline analysis of the EEG data from both systems was performed using BrainVision Analyzer 2 (Brain Products, 2012). The Brain Products data were recorded in a format compatible with BrainVision. Digital event markers were integrated and correctly numbered to reflect different event types (congruent, incongruent, and neutral stimuli). Correct and incorrect responses were marked using participant response data extracted from Eprime and a Perl script that was previously prepared and used with the task.

EEG data recorded with Mobita were exported to EDF format (Kemp et al., 1992) and imported to BrainVision Analyzer. As a result of the bespoke solution for digital signal recording, only the values of 0 and 1 could be registered to mark events. Therefore, all stimuli events were marked when a change from 0 to 1 occurred in the digital channel and all responses were marked when 1 changed back to 0. In R Studio (RStudio Team, 2020), task-relevant data recorded with Eprime were used alongside the Acqknowledge markers to label the type of condition and correct/incorrect responses.

For a detailed description of the preparation of digital markers for the bespoke digital signal transfer used for Mobita in this study, see the Supplementary Material. One particularly significant difficulty observed during this process was data loss in the Mobita recordings of three participants caused by two types of signal drops. The first type was caused by Wi-Fi connection issues and led to no data being recorded (all channels were flat) for the duration of a few trials at each instance. The second type of signal drop led to a complete termination of recording in the Acqknowledge software for about 1 min in one case. The cause of this is unknown. Acqknowledge does not record the duration of recording termination in such situations, and this had to be determined manually by comparing the timings recorded by Acqknowledge and Eprime and determining the temporal location of the gap. Data loss in this case included 68.09 s of data (40 consecutive trials).

#### Pre-processing

For pre-processing, only channels overlapping between the two systems were selected. In the Brain Products setup, TP9 and TP10 and in Mobita, Fpz and Oz were excluded. Data were visually inspected for channels with no or extreme activity. No channels were interpolated in the Brain Products recordings. In Mobita, channels were interpolated in two recordings (one channel in the first case and two channels in the second case). In addition, during the inspection of the Mobita data, the mastoid channels were observed to be extremely noisy or flat in three recordings. Therefore, all EEG recordings from both systems were re-referenced to the average activity of the subset of overlapping channels. For the Brain Products setup, this included the initial implicit reference but in Mobita, the mastoid electrodes were excluded. Subsequently, the following filters were applied: 0.1 Hz high-pass, 50 Hz low-pass and 50 Hz notch filter with threshold selection designed to avoid ERP distortion and ensure the most optimal signal-to-noise levels based on best practice recommendations and previous EEG system comparisons (Tanner et al., 2015, 2016). Data were then re-sampled to 512 Hz in both systems. Before artefact cleaning, all non-task data were removed. This included the start and the end of the recordings as well as breaks between the blocks leaving only task-related block segments for further analysis.

Ocular correction independent component analysis (ICA) was used with default BrainVision Analyzer settings (Brain Products GmbH, 2019: 279) to automatically detect components around blinks. Channels used to train the algorithm were HEOG and VEOG in the Brain Products setup and Fp1 and F7 in Mobita. Component rejection was semi-automatic where one researcher (M.T.) inspected each ICA component and confirmed its removal/retainment. There were no significant differences between the number of components removed in the Brain Products (*Median* = 2.5, *IQR* = 1) and Mobita systems (*Median* *=* 2.5, *IQR* *=* 3; *V* *=* 7.5, *p* *=* 0.59). Data were epoched into two types including stimulus-locked epochs for frequency and P300 analyses and response-locked epochs with correct and incorrect responses for ERN analyses. Stimulus-locked epochs were selected at −200 to 500 ms respective to stimulus onset. Response-locked epochs were selected at −150 and 200 ms respective to response onset. Automatic artefact rejection was performed on all epochs using the default settings of BrainVision Analyzer excluding trials with activity below 0.5 μV for a duration of 50 ms, amplitude values falling outside of the −200 and 200 μV range, absolute amplitude difference above 200 μV in any interval of 200 ms and finally, voltage steps of more than 50 μV per millisecond. The number of rejected artefactual epochs is included as one of measures of noise and reported in the ‘Results’ section.

#### Noise measurements

To assess potential noise in raw data, the fast Fourier transform was applied to unfiltered data that were re-referenced, re-sampled and segmented to task-related blocks without any ocular correction or artefact rejection. 0.1–2 Hz and 49–51 Hz power values were then extracted for further analysis to understand the potential of slow drift interference (de Cheveigné and Arzounian, 2018) and line noise interference (Leske and Dalal, 2019).

Levels of noise were also assessed in the fully pre-processed and cleaned stimulus-locked data. First, the fast Fourier transform was applied and power values at 0.1–2 Hz and 49–51 Hz were extracted for a before-and-after pre-processing comparison of noise. In addition, the proportion of rejected artefactual stimulus-locked trials was calculated for both systems. Next, SNR and the root mean square (RMS) values were calculated from a subset of electrodes excluding those located at the edges of the cap which are particularly prone to noise. The remaining subset therefore included F3, Fz, F4, FC5, FC1, FC2, FC6, C3, Cz, C4, CP5, CP1, CP2, CP6, P3, Pz and P4. SNR and RMS metrics are common in studies comparing different types of EEG equipment (Kam et al., 2019; Mathewson et al., 2017; Oliveira et al., 2016). SNR was calculated from averaged, stimulus-locked trials using the formula embedded within the BrainVision Analyzer software (Brain Products GmbH, 2019: 402) which estimates the average signal power as squared absolute values of the average data across all data points and all frequency bins while noise power is estimated as the biased variance of the data across all segments. The values were extracted for all electrodes included in the specified subset and then averaged for each participant. RMS values were determined with BrainVision Analyzer’s RMS function which calculated the root from the average of the squares of the individual values (Brain Products GmbH, 2019: 210) within the stimulus-locked epochs’ baseline period of −200 to −100 ms prior to stimulus onset.

#### Time-frequency measurements

For time-frequency analyses of task-related brain activity, the fast Fourier transform was applied to the pre-processed stimulus-locked epochs. The number of epochs was matched between the systems for each participant with an average of 467.8 (*SD* *=* 90.58) epochs per participant. Power was extracted from the same subset of electrodes used in the SNR and RMS analyses (see previous section). The data were analysed in four frequency bands including theta (4–8 Hz), alpha (8–14 Hz), low beta (14–24 Hz) and high beta (24–30 Hz). For the comparison of power activity within these bands, we followed the method used by Kam et al. (2019) whereby five electrodes with maximum activity were identified per system and power was averaged across the overlapping channels. For theta and alpha, electrodes with the most positive power were selected (theta Fz, F4, FC1, FC2, alpha Fz, Pz, P4) due to expected engagement of cognitive control and attentional processes. For low and high beta, electrodes with the least positive power were selected (low beta CP1, CP2, Pz, high beta CP1, CP2, Pz, P4) as pre-response motor-related beta desynchronisation was expected (Doyle et al., 2005).

#### ERP measurements

For ERP analyses, baseline correction was applied to all fully pre-processed epochs. The baseline window was located at −200 and −100 ms prior to stimulus onset for stimulus-locked P300 epochs and at −150 to −100 ms for response-locked ERN epochs following best practice recommendations by Alday (2019). In P300 analyses, the same epochs were used as those in time-frequency analyses. ERN epochs were also matched between the two systems and the number of epochs for correct and error responses was matched as well. The selection of trials for matching was based on the order of occurrence. There was an average of 27.5 (*SD* *=* 18.93) trials per participant. There was one participant with only five error trials available for ERN analyses. This is below the recommended value of at least six trials (Olvet and Hajcak, 2009) so this case was removed from all ERN analyses. EEG activity was then averaged across trials. Mean amplitude for the P300 component was extracted at a 300–500 ms interval at Pz and for the ERN component at a 0–100 ms interval at Cz which is suitable for flanker task analyses (Klawohn et al., 2020; Rietdijk et al., 2014). To investigate the characteristics of the obtained ERPs, peak amplitude and latency were also extracted. ERN and P300 peaks were semi-automatically identified in BrainVision Analyzer.

#### Statistical analyses

All dependent variables were tested using the Wilcoxon test to statistically compare within-subject medians (Fagerland and Sandvik, 2009) and the Fligner-Killeen test to assess homogeneity of variance (Michael and Edwardes, 2001) between the two systems. The dependent variables were divided into the following groups for the purpose of controlling the familywise error: average power values at 0.1–2 Hz and 49–51 Hz before and after pre-processing; proportion of artefactual stimulus-locked trials and stimulus-locked noise level metrics including SNR and RMS; averaged power for four frequency bands (theta, alpha, low beta, high beta); mean amplitude, peak amplitude and peak latency for P300 and ERN. Bonferroni correction was applied accordingly.

##### Exploratory analyses

Due to the observed P300 and ERN topographical differences between the two systems, we decided to explore whether SNR values might differ between the two systems by electrode locations. We therefore divided the SNR electrode subset into three general regions: frontal (Fp1, F2p, F7, F3, Fz, F4, F8), central (FC5, FC1, FC2, FC6, C3, Cz, C4, CP5, CP1, CP2, CP6) and posterior (P7, P3, Pz, P4, P8, O1, O2). We calculated the average SNR values for these regions for each participant and each system and compared these values using the Wilcoxon and Fligner-Killeen tests. Bonferroni correction was applied.

##### Power analysis

The results of the study showed some significant differences between the EEG systems and also a number of potentially practically informative non-significant differences with large effect sizes. This could be due to the small sample size, the large number of statistical tests and the Bonferroni correction for multiple comparisons. To help in the interpretation of these potentially informative effects, we decided to run a post hoc power analysis on the smallest large effect size obtained. Post hoc power analyses are discouraged when reliant on effect sizes achieved with a limited sample as it is not possible to estimate whether these effect sizes reflect the true population effects (Lakens, 2021). However, considering the methodological focus of the current study and the practical importance of differences between the two systems, it is important to understand the current study’s statistical sensitivity to detect the effects of interest. The power calculation was conducted with G*Power (Faul et al., 2007) using the results of the 0.1–2 Hz raw data power comparison between the Brain Products setup and Mobita where a non-significant large effect size was found (*r* *=* 0.53). The analysis yielded power of 21.7% (calculation output https://osf.io/fdmzx/). This therefore reflects very small chances for obtaining statistically significant results, even for large effect sizes of interest with the current sample and with the given number of tests and comparisons. We therefore implemented the Bayes factor calculation in favour of the alternative hypothesis (BF_10_, further referred to as BF) for all Wilcoxon tests to help with the interpretation of non-significant results (Dienes, 2014). The prior was set to the jmv’s R package default of 0.7 (function ttestPS()) to reflect our expectations that differences between the two systems were likely. BF values of ⩽0.33 were deemed as evidence in support of no statistical difference and ⩾3 as evidence in support of statistical difference between the systems.

## Results

### Researcher and participant experience

For the fit of the cap, during the Brain Products setup recording, common comments referred to the chin strap that felt ‘scratchy’ for some participants. It had to be adjusted throughout the procedure to improve comfort. During the Mobita recording, in some instances the front of the cap put pressure on the forehead which led to moderate discomfort. The Mobita cap has a tightening string which helps to adjust the fit though it is positioned around the face only. We either loosened it up for participants or refrained from using it to improve comfort.

Participants were generally impressed with Mobita due to its shorter preparation time. The preparation procedure that involved participants took up to 30 min compared with up to an hour for the Brain Products setup. Some were also relieved that they did not have to wash their hair following the procedure and could quickly go back to their activities after participating in the study. One participant mentioned that they only agreed to participate again when they were informed that the procedure would be shorter this time and no gel would be used. It is worth noting, however, that from the experimenter point of view, the total time taken to prepare each system was not very different as Mobita required extra preparation before participant arrival.

### Noise comparisons

**Figure 2.**
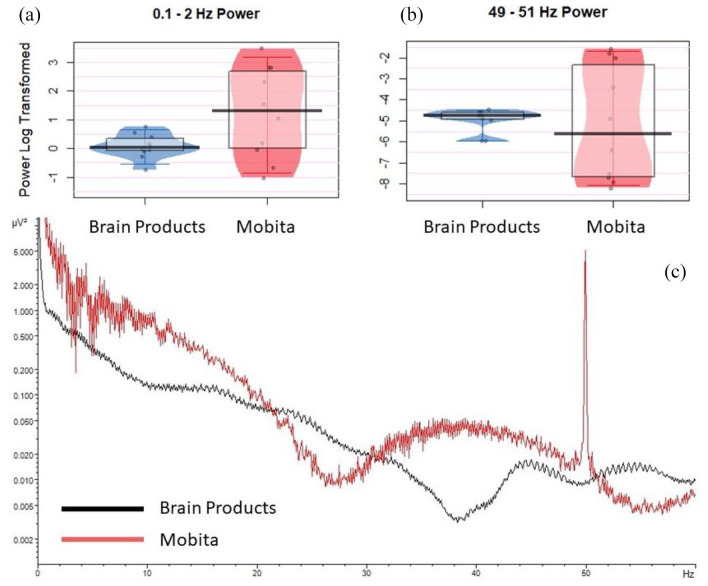
Raw data average power prior to pre-processing. Jittered individual data points are plotted for (a) 0.1–2 Hz and (b) 49–51 Hz to compare between the Brain Products and Mobita recordings. The vertical bar marks the median and the shaded box reflects the interquartile range. (c) A representation of log-transformed power spectrum at 0–60 Hz for Brain Products and Mobita.

Prior to pre-processing, average power was significantly more variable for Mobita at 0.1–2 Hz (χ^2^ = 8.9, *p* = 0.022) and 49–51 Hz (χ^2^ = 11.0, *p* = 0.007) compared to the Brain Products setup, but there were no significant differences for median comparisons. In fact, the BF obtained for the difference at 49–51 Hz was 0.32 suggesting that the two systems picked up a statistically comparable level of line noise. Figure 2 displays individual data plots with average power at 0.1–2 Hz and 49–51 Hz for both systems as well as the overall averaged power spectrum for the raw EEG data.

**Figure 3.**
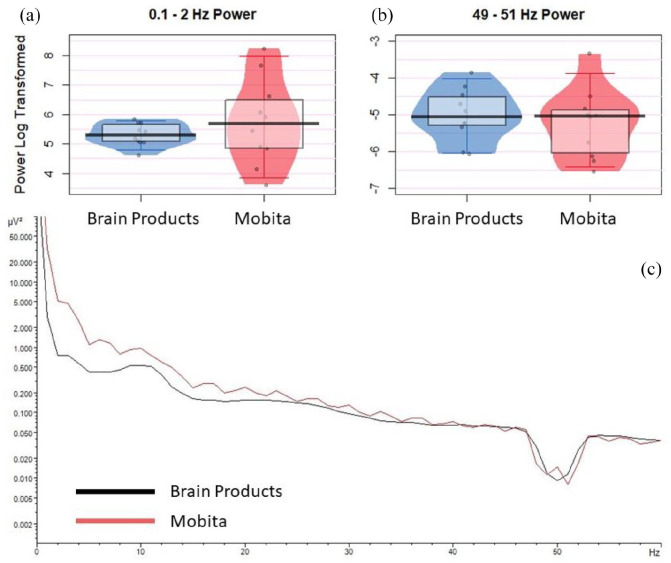
Pre-processed data average power. Jittered individual data points are plotted for (a) 0.1–2 Hz and (b) 49–51 Hz to compare between the Brain Products and Mobita recordings. The vertical bar marks the median and the shaded box reflects the interquartile range. (c) A representation of log-transformed power spectrum at 0–60 Hz for Brain Products and Mobita.

Following pre-processing, there were no statistically significant median or variance differences between the two systems. The BF values were inconclusive; thus, there is no evidence to suggest that the two systems are comparable despite the lack of significant differences. Figure 3 displays individual data plots with average power at 0.1–2 Hz and 49–51 Hz for both systems as well as the overall averaged power spectrum for the pre-processed EEG data. Exact statistical results are presented in Table 2.

**Table 2.** Median and interquartile ranges displayed for both systems before and after pre-processing for average power at 0.1–2 Hz and 49–51 Hz.

	Brain Products	Mobita	Median	BF	Variance
			Wilcoxon		Fligner-Killeen
	Median (IQR)		*V, p (r)*		χ^2^, *p*
Raw data					
0.1–2 Hz	0.03 (0.42)	1.31 (2.67)	11, 0.84 (0.53)	1.37	8.90, 0.022*
49–51 Hz	−4.76 (0.33)	−5.64 (5.30)	30, 1.0 (0.08)	**0.32**	11.01, 0.007*
Pre-processed data					
0.1–2 Hz	5.31 (0.57)	5.68 (1.61)	18, 1.0 (0.31)	0.42	6.86, 0.07
49–51 Hz	−5.06 (0.78)	−5.05(1.16)	32, 1.0 (0.21)	0.37	0.44, 1.0

BF: Bayes factor; IQR: interquartile range.

The results of statistical comparisons of medians (Wilcoxon) and variance (Fligner-Killeen) are also presented. Significant differences are marked with an asterisk. Bayes factors suggesting no significant differences are reported in bold. Bonferroni correction was used to adjust the obtained *p*-values.

**Figure 4.**
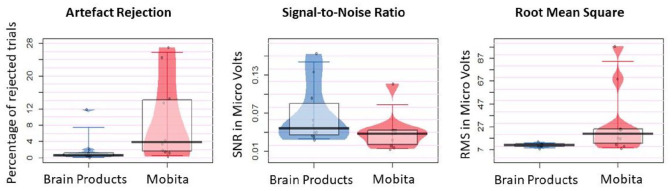
Jittered individual data points reflect average percentage of rejected artefactual trials, signal-to-noise ratio and root mean square for Brain Products and Mobita recordings. The vertical bar marks the median and the shaded box reflects the interquartile range.

The artefact rejection rate for Mobita was significantly higher (*V* *=* 1, *p* *=* 0.008, *r* *=* 0.85) and more variable across participants (χ^2^ = 6.42, *p* *=* 0.023) than in the Brain Products setup. There were no statistically significant differences between the two systems in terms of SNR medians or variance but the obtained BF suggests that the two systems are likely statistically different. Baseline RMS was significantly more variable for Mobita compared with the Brain Products setup (χ^2^ = 12.74, *p* *=* 0.001) but there was no median difference, and the BF was inconclusive. Exact statistical results are reported in Table 3, and individual data plots are presented in Figure 4.

**Table 3.** Median and interquartile ranges displayed for both systems for all three measures of noise: percentage of rejected artefactual trials, signal-to-noise ratio, and root mean square.

	Brain Products	Mobita	Median	BF	Variance
			Wilcoxon		Fligner-Killeen
	Median (IQR)		*V, p (r)*		χ^2^, *p*
Artefact rejection %	0.58 (0.64)	3.80 (12.60)	1, 0.008 (0.85)*	1.9	6.42, 0.023*
SNR (μV)	0.05 (0.05)	0.04 (0.04)	48, 0.15 (0.66)	3.2*	1.67, 0.76
RMS (μV)	10.80 (1.83)	20.80 (12.39)	5, 0.08 (0.73)	1.5	12.74, 0.001*

BF: Bayes factor; IQR: interquartile range; SNR: signal-to-noise ratio; RMS: root mean square.

The results of statistical comparisons of median (Wilcoxon) and variance (Fligner-Killeen) are also presented. Significant differences are marked with an asterisk. Bonferroni correction was used to adjust the *p*-values.

### Frequency power comparisons

**Figure 5.**
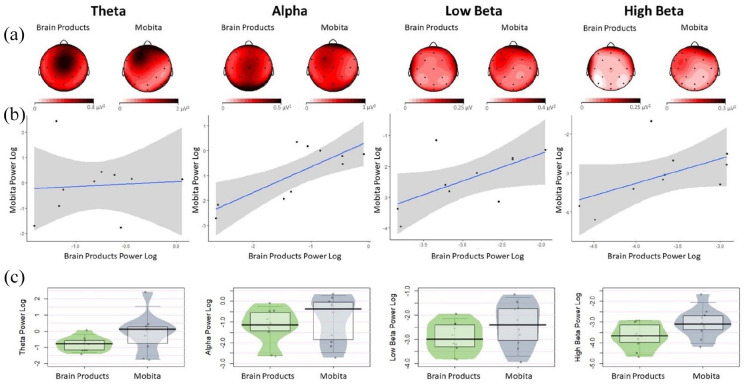
(a) Topographies for all power frequencies are displayed for comparison between the two systems. The topographies have not been normalised and different scales are used for Brain Products and Mobita. (b) Scatter plots with fitted line of best fit and confidence intervals to visually reflect the relationship between power obtained with the Brain Products and Mobita systems. (c) Jittered individual data points reflecting average power for each participant recorded with each system. The vertical bar marks the median and the shaded box reflects the interquartile range.

Frequency power was compared between the Brain Products setup and Mobita across four bands (theta, alpha, low beta and high beta). No statistically significant results were obtained for the median and variance tests of difference. However, the BF reflects a statistically likely difference between the two systems for the high beta frequency. Medians, interquartile ranges and exact test results can be found in Table 4. Figure 5 displays topographical power distribution, scatter plots and individual data plots for the Brain Products setup and Mobita across the four frequency bands.

**Table 4.** Median and interquartile ranges displayed for both systems for four frequency bands: theta, alpha, low beta and high beta.

	Brain Products	Mobita	Median	BF	Variance
			Wilcoxon		Fligner-Killeen
	Median (IQR)		*V, p (r)*		χ^2^, *p*
Theta	−0.78 (0.59)	0.10 (1.03)	12, 1.0 (0.50)	0.95	1.03, 1.0
Alpha	−1.15 (0.88)	−0.38 (1.82)	18, 1.0 (0.31)	0.79	1.23, 1.0
Low beta	−2.35 (0.67)	−1.65 (0.82)	6, 0.22 (0.69)	1.91	1.72, 1.0
High beta	−3.67 (0.84)	−3.11 (0.67)	3, 0.08, (0.79)	4.69*	0.07, 1.0

BF: Bayes factor; IQR: interquartile range.

The results of statistical comparisons of medians (Wilcoxon) and variance (Fligner-Killeen) between the two systems are also displayed. Significant differences are marked with an asterisk. Bonferroni correction was used to adjust the obtained *p*-values.

### ERP comparisons

#### P300

**Figure 6.**
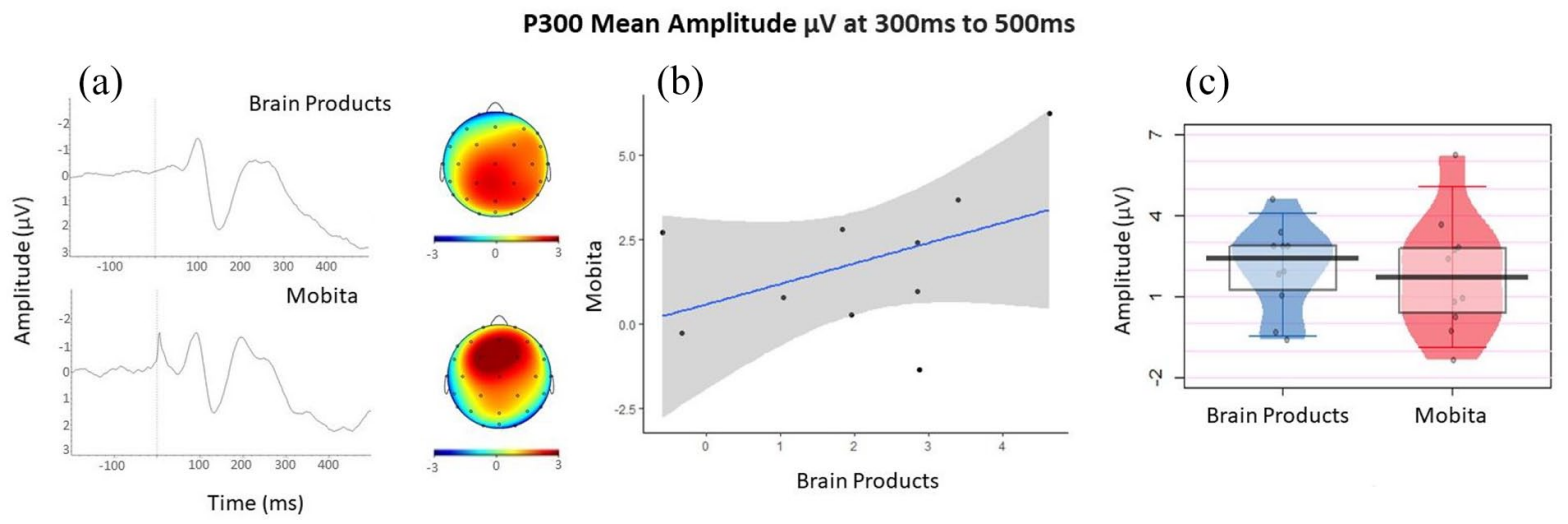
(a) The P300 waveforms and topographies for each system. (b) Mean amplitude scatter plot with a fitted line of best fit and confidence intervals to visually reflect the relationship between the Brain Products and Mobita systems. (c) Jittered individual data points reflecting average mean amplitude for each participant recorded with each system. The vertical bar marks the median and the shaded box reflects the interquartile range.

No statistically significant differences were found between Brain Products and Mobita systems for the mean amplitude, peak latency and peak amplitude of the P300 component at 300–500 ms in terms of medians and variance which is confirmed by the obtained BF values for all comparisons. Table 5 displays the medians and interquartile ranges observed, as well as exact statistical results. Figure 6 displays the P300 waveforms, topographies, a scatter plot and an individual data plot for comparison of P300 mean amplitude values.

**Table 5.** Median and interquartile ranges are displayed for both systems for the measures of P300 mean amplitude at 300–500 ms, ERN mean amplitude at 0–100 ms, peak amplitude values and peak latency.

	Brain Products	Mobita	Median	BF	Variance
			Wilcoxon		Fligner-Killeen
	Median (IQR)		*V, p (r)*		χ^2^, *p*
P300					
Mean amplitude (μV)	2.40 (1.63)	1.69 (2.39)	31, 1.0 (0.11)	**0.33**	1.53, 1.0
Peak amplitude (μV)	2.54 (1.75)	2.49 (3.22)	21, 1.0 (0.15)	**0.33**	1.69, 1.0
Peak latency (ms)	346 (38)	343 (67)	23, 1.0 (0.16)	**0.32**	2.85, 1.0
ERN					
Mean amplitude (μV)	−2.69 (5.46)	−0.89 (1.62)	1, 0.09 (0.85)	6.96*	1.04, 1.0
Peak amplitude (μV)	−3.96 (7.96)	−4.56 (0.98)	36, 1.0 (0.02)	**0.32**	0.31, 1.0
Peak latency (s)	41 (16)	6 (29)	13, 0.17 (0.87)	17.3*	1.48, 1.0

BF: Bayes factor; IQR: interquartile range; ERN: error-related negativity.

The results of statistical comparisons of medians (Wilcoxon) and variance (Fligner-Killeen) are displayed for all measures. Significant differences are marked with an asterisk. Bayes factors suggesting no significant differences are reported in bold. Bonferroni correction was used to adjust all obtained *p*-values.

#### ERN

No statistically significant differences were found between Brain Products and Mobita systems for the mean ERN amplitude at 0–100 ms in terms of medians and variance. In addition, no statistically significant differences in medians or variance were identified in ERN peak amplitude or peak latency. However, the BF values suggest that while the two systems are comparable in terms of ERN peak amplitude, the mean amplitude and latency could be considered statistically different. In Figure 6(a), it is evident that the ERN peak occurs early in Mobita, almost directly at the time of response onset. Table 5 displays the medians and interquartile ranges observed, as well as exact statistical results. Figure 6 displays the P300 waveforms, topographies, a scatter plot and an individual data plot for comparison of ERN mean amplitude values.

### Exploratory analysis

**Figure 7.**
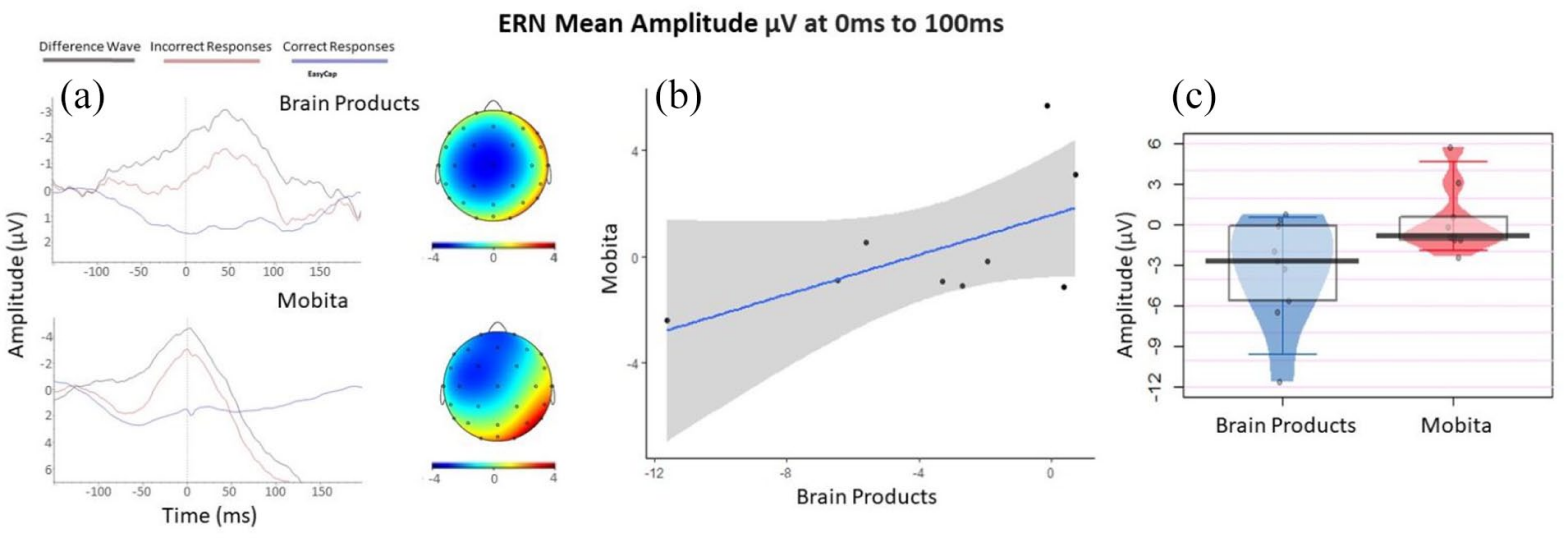
(a) The ERN waveforms and topographies for each system. (b) Mean amplitude scatter plot with a fitted line of best fit and confidence intervals to visually reflect the relationship between Brain Products and Mobita systems. (c) Jittered individual data points reflecting average mean amplitude for each participant recorded with each system. The vertical bar marks the median and the shaded box reflects the interquartile range.

Exploratory analysis focused on the differences in SNR values by electrode locations including frontal, central and posterior. In Figures 6(a) and 7(a), we have observed that the obtained ERP peaks are shifted frontally. The shift is especially prominent for P300 (Figure 6(a)). We were therefore interested in finding out whether some electrodes might be particularly susceptible to high noise levels. We found no differences in variance between the two systems. The median difference was only significant for posterior electrodes and this was further confirmed by the obtained BF. Table 6 displays the medians and interquartile ranges observed, as well as exact statistical results. Figure 7 presents the individual data plot for the comparison of SNR values across electrode location and between Brain Products and Mobita systems.

**Table 6.** Median and interquartile ranges displayed for both systems for SNR values recorded with frontal, central and posterior electrodes.

SNR (μV)	Brain Products	Mobita	Median	BF	Variance
			Wilcoxon		Fligner-Killeen
	Median (IQR)		*V, p (r)*		χ^2^, *p*
Frontal electrodes	0.05 (0.05)	0.03 (0.02)	47, 0.49 (0.63)	2.00	0.46, 1.0
Central electrodes	0.04 (0.05)	0.03 (0.02)	46, 0.63 (0.60)	1.96	0.33, 1.0
Posterior electrodes	0.07 (0.04)	0.03 (0.03)	54, 0.04 (0.85)*	32.44*	0.09, 1.0

BF: Bayes factor; IQR: interquartile range.

The results of statistical comparisons of medians (Wilcoxon) and variance (Fligner-Killeen) are provided. Statistically significant results are marked with an asterisk. Bonferroni correction was used to adjust all obtained *p*-values.

## Discussion

Mobita is an example of an innovative approach to EEG recordings via water-based electrodes and a wireless setup. These relatively new systems may be of interest to researchers who want to shorten the EEG preparation time (e.g. for children or clinical populations), reduce the possibility of sensory discomfort (e.g. for participants with sensory sensitivities) or considering independent recordings taken by participants or patients at home. From our experience, Mobita is currently not suited for a quick and easy application in studies aiming to analyse EEG recordings in time-locked epochs for frequency or ERP comparisons. Researchers considering the use of such systems should weigh the potential benefits against technical, practical and data quality disadvantages presented in this study.

### Technical and practical considerations

Participants in the current study had a generally positive experience when using Mobita and some expressed their preference for Mobita over the Brain Products setup due to the reduced preparation time and not having to wash their hair following the procedure. However, none of the participants had any pre-existing sensory sensitivities, hyperactivity or attention difficulties. It is not clear if Mobita would be more beneficial for participants with such difficulties and how much improvement it could bring overall to the experience of the EEG procedure.

From the researcher point of view, however, the Brain Products setup was more optimal in terms of the technical and practical application while the Mobita system required more adaptations and time-consuming solutions at all stages – setup, recording and analysis. At setup, it required a bespoke solution to allow for it to record synchronised digital and EEG signals for time-locked analyses. This is despite the setup being marketed as being able to record ERPs with no modifications or solutions that were alternative to the original expectations. The initial process of setting up Mobita is not straightforward and may require technicians or engineer assistance. At recording, the Mobita cap was not well fitted for some participants as one adjustable size was used instead of using caps fitted for individual head sizes like in the case of the Brain Products setup. It was also problematic that the mastoid reference electrodes were embedded within the cap as it was difficult to keep them close to the scalp to obtain good quality reference data. As a result, an average reference was used during pre-processing instead. In addition, the unavailability of an electrode impedance measure meant that the researcher could not easily check and compare signal quality across the cap. Therefore, this increased the chances for Mobita EEG recordings to register more noise than the Brain Products setup. Mobita was also susceptible to signal drop and recording termination which led to loss of data. During the analysis, the digital marker signal for cognitive task events (stimuli and responses) had to be extracted from Acqknowledge and labelled (condition and response types) externally while during the Brain Products recording the digital signal was mostly already labelled into different types. This was especially challenging for instances when the EEG and digital signal drops occurred as the gaps had to be manually detected and the markers were then realigned. However, this could be mitigated by choosing an option to record the signal directly to the logger instead of transferring the data wirelessly to the computer for recording. See the recommendation section below.

### Data noise

Mobita had higher variance in registered noise at 0.1–2 Hz and 49–51 Hz in raw data compared to the Brain Products setup suggesting that signal quality in Mobita is more likely to be affected by individual factors such as environmental condition, head shape and cap fit. Following pre-processing, power at 49–51 Hz became visually comparable with Brain Products (Figure 2). However, this was not the case for power at 0.1–2 Hz, and power variance only reduced slightly numerically (Table 2, Figure 3(a)). From a practical perspective, this may prompt researchers who are using the system in the future to consider the potential likelihood of slow drifts in Mobita recordings even after standard pre-processing steps have been applied. These drifts may be caused by poor electrode-to-skin contact and may mask slow cortical activity in studies looking at low frequencies or distort ERP components (de Cheveigné and Arzounian, 2018). In addition, we observed statistically significant median and variance differences in the artefact rejection rates between the systems indicating higher noise levels and further data loss following the pre-processing procedures. SNR was generally lower for Mobita with the BF value indicating that this was a substantial statistical difference. Median comparison of SNR values per channel area (frontal, central and parietal) reached statistical significance for posterior electrodes which was further confirmed with the BF value. The RMS values were significantly more variable for Mobita. Taken together, these findings indicate that Mobita registers more noise at recording which can be mostly improved with pre-processing. However, the high artefact rejection rates, low SNR and variable RMS suggest that the data will likely still contain higher levels of noise compared to standard EEG systems. This may affect the EEG results as explained below.

### EEG results

**Figure 8.**
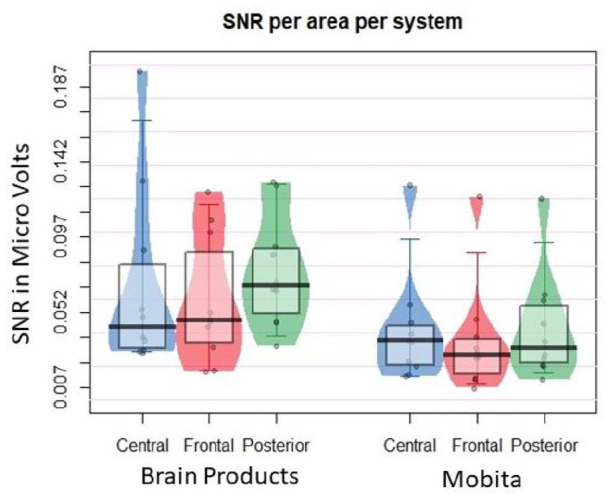
Jittered individual data points reflecting average SNR values for each participant presented by electrode location and recording system. The vertical bar marks the median and the shaded box reflects the interquartile range.

Regarding the frequency analyses, power across the four bands (theta, alpha, low beta and high beta) did not significantly differ between the two systems in terms of median or variance comparisons. However, the obtained BF for high beta frequency suggests that the two systems are statistically different. For this frequency, we expected to observe motor-related beta desynchronisation in the parietal regions reflected with power values that are negative or close to 0. Mobita activity seemed to be more positive than Brain Products setup activity potentially masking the motor-related beta desynchronisation. This might have been caused by significantly lower SNR in the parietal channels in the Mobita system as evident from the exploratory analysis (Figure 8). In addition, from a visual comparison of the scatter plots for all four frequency bands, theta power is the least consistent between the two systems. This could be due to the observed increase in slow drift noise in Mobita (Figure 3). The topographies for all bands are relatively similar based on visual comparisons with a slight frontal shift of maximal power observed in theta and alpha bands recorded with Mobita.

With regards to the ERP analyses, statistical comparisons between the two systems were non-significant for P300 mean amplitude, peak amplitude and peak latency. In fact, the obtained BF suggests that the two systems are not statistically different in terms of these measures. Therefore, there is no indication from the current data that Mobita could yield unreliable P300 results. The P300 waveforms seemed visually similar. However, looking at the P300 topographies (Figure 6(a)), the Brain Products setup recorded maximal P300 activity over parietal regions which is expected in a Flanker task (Klawohn et al., 2020). In contrast, the P300 activity was maximal over the frontal region in Mobita which is unexpected. The topographies should be visually similar as the same participants were tested on the same task with both systems. Although we cannot rule out the possibility that this frontal shift could have been caused by practice effects, several previous studies showed a reduction of the P300 amplitude and a more parietal shift of the component as a result of practice effects (Friedman et al., 2001; Nakata et al., 2015). As in the case of beta power, it is likely that the posterior activity was masked by low SNR in parietal and centro-parietal channels which could have caused the shift of maximal P300 activity more frontally in Mobita. This is a significant issue for consideration in future research as it may lead to false interpretation of results which may be inconsistent with previous literature and the current understanding of frontal and parietal P300 variants (van Dinteren et al., 2014).

For ERN mean amplitude, peak amplitude and peak latency, there were no statistically significant differences between the two systems. However, the BF values showed the two systems to be statistically comparable in terms of ERN peak amplitude but different in terms of mean amplitude and peak latency. The latency shift is also evident by visually inspecting the ERN waveform (Figure 7(a)). The Mobita ERN peak occurs almost at the onset of response, but it is normally expected at around 50 ms post response onset in Flanker tasks (Klawohn et al., 2020; Riesel et al., 2013) which is accurately reflected in the Brain Products waveform. The likely explanation for this latency shift is the bespoke solution for digital marker recording used in Mobita. It seems that stimulus-locked events can be accurately marked with the current system where the digital signal is set from 0 to 1 at stimulus onset since the peak latency for the P300 component did not differ between the two systems. At response onset, the digital signal is set back from 1 to 0 and it seems that this event was recorded approximately 35 ms earlier than the actual response if we directly compare the Brain Products and Mobita peak latency median values (Table 5). This issue may again have adverse consequences in future research and lead to false interpretation of results that will not be consistent with the current knowledge about the ERN. Otherwise, visual inspection of the ERN waveforms suggests that the ERN component registered with both systems is rather similar and occurs to be slightly clearer for Mobita than Brain Products. This could potentially be due to practice effects as all Mobita recordings were taken during phase 2 of the study. The topographies are also visually similar with a slight frontal shift observed in Mobita, but this is unlikely to lead to false interpretation.

### Limitations

As the Mobita recording was acquired after the Brain products recording for all participants, on the same task, there could be a confound of order and practice effects. These could potentially have influenced the observed topographical, ERP and power differences. Previous reports suggest that to reduce the impact of practice effects, re-testing should be done later than within 3 months and with no additional repetitions of the task in between (Bartels et al., 2010; Hausknecht et al., 2007). In the current study, participants completed the task only twice with a 7–12 months interval, thereby minimising potential practice effects. Another confounding factor could be the fact that the gel-based system was recorded in a sound-proofed room, while the water-based system was not. This may have led to higher participant distraction in Mobita recordings as well as reduced SNR (Ledwidge et al., 2018).

One limitation of the current study is the small sample size and low statistical power. However, considering the practical nature of the study, even results from a limited sample can be highly informative for the development of future protocols. The observed patterns and differences between the two systems will help researchers to understand the possible pitfalls of the system so that preventive solutions can be developed. For instance, when measuring P300, special attention should be given to ensuring good quality signal in the parietal electrodes which are susceptible to noise. These results can also help to explain unexpected results that researchers might obtain in the future due to noisy recordings.

Another limitation of the study is that in case of Mobita, it was the first time that the system was used to collect and analyse EEG data at the School of Psychology, University of Surrey. The Brain Products system has been used numerous times and best practices have already been established over the years. It is therefore possible that data recorded with the Brain Products setup were of better quality because the researchers were able to use it more confidently. It is possible that with the development of best practice solutions as well as further practice in the use of water-based systems, the recorded data will also be of higher quality. The current study is an important step in the development of these best practice solutions.

Finally, authors have not conducted any formal recordings of the objective experiences at data acquisition for participants nor themselves. The experiences were retrieved from lab notes and memory. However, all described experiences illustrate difficulties which have led to certain practical adaptations or methodological considerations for the future as detailed in the section below. Therefore, the subjective nature of these experiences presents a case scenario of a real-life application of a water-based EEG system.

### Recommendations for future research

Based on the results presented in this study, we provide a set of general recommendations to avoid technical difficulties and false conclusions in studies using water-based electrode EEG systems. For more specific explanations regarding the assessment of electrode noise prior to recording, the bespoke solution on digital event markers and event markers labelling in the current study, see the Supplementary File.

For studies using water-based EEG systems for low-frequency effects and ERPs, researchers may want to consider robust solutions for detrending data in order to prevent the slow drifts from distorting or masking the effects of interest. For studies focused on effects located in parietal regions, such as the P300 or beta desynchronisation, researchers should be especially careful about ensuring low noise levels. They should plan for regular assessments of data quality and perhaps consider taking more than one recording during a single procedure. The power spectrum of all channels can be assessed for noise during the recording breaks and the electrode fitting can be re-adjusted to improve the electrode to scalp contact. This would be especially beneficial for long procedures to ensure that the electrodes have not become dry or dislocated.

For studies requiring the use of mastoid reference channels, researchers should consider the likelihood of high noise levels due to the difficulty in sustaining good electrode to skin contact. One solution is to apply a bandage or an elastic band to secure the electrodes in place (see the Supplementary File for an example).

Researchers should take a careful approach if their studies require good temporal precision (e.g. in ERP analyses) and if they are using a new EEG system which may or may not have been designed for time-locked analyses. To avoid possible latency shifts (as observed in this study), one solution is to synchronise the EEG recording with the stimulus presentation software at the point of the first stimulus onset and use event timing values from the stimulus recording software output rather than the digital signal.

Finally, to avoid data loss due to signal drops in wireless systems, we recommend that researchers record EEG signal directly to the data logger instead of sending the data wirelessly to a recording computer. Issues associated with data loss and high noise levels can be further alleviated by aiming to recruit larger samples and increasing the number of trials in tasks to preserve statistical power. It may also help to reduce data noise if recordings are performed in sound-proofed rooms rather than naturalistic environments that are common in wireless recordings. Another consideration for the wireless setup is the difficulty in sending triggers (currently only possible with a wired connection).

## Conclusion

Water-based EEG systems could potentially help to reduce participant involvement time and discomfort. However, they may require a number of time-consuming adaptations which are not necessary when using the state-of-the-art gel-based systems. Researchers should be aware of the likelihood that water-based systems will register high levels of noise which may affect analyses investigating ERPs, low frequencies and parietal activity. Otherwise, they may be at risk of drawing wrong conclusions from their results.

## Supplemental Material

sj-docx-1-bna-10.1177_23982128211053698 – Supplemental material for In search for the most optimal EEG method: A practical evaluation of a water-based electrode EEG system Click here for additional data file. Supplemental material, sj-docx-1-bna-10.1177_23982128211053698 for In search for the most optimal EEG method: A practical evaluation of a water-based electrode EEG system by Marta Topor, Bertram Opitz and Philip J. A. Dean in Brain and Neuroscience Advances

## References

[bibr1-23982128211053698] Alday PM (2019) How much baseline correction do we need in ERP research? Extended GLM model can replace baseline correction while lifting its limits. Psychophysiology 56(12): e13451. 10.1111/psyp.13451 31403187

[bibr2-23982128211053698] Bartels C Wegrzyn M Wiedl A , et al. (2010) Practice effects in healthy adults: A longitudinal study on frequent repetitive cognitive testing. BMC Neuroscience 11(1): 118. 20846444 10.1186/1471-2202-11-118 PMC2955045

[bibr3-23982128211053698] Biopac Systems Inc (2016) How should I prepare electrode sites? Available at: https://www.biopac.com/knowledge-base/how-should-i-prepare-electrode-sites/ (accessed 11 March 2021).

[bibr4-23982128211053698] Biopac Systems Inc (2018) Acqknowledge 5.0.3. Goleta, CA: Biopac Systems.

[bibr5-23982128211053698] Biopac Systems Inc (2019) Mobita^®^ user manual. Available at: https://www.biopac.com/wp-content/uploads/Doc85.pdf (accessed 11 March 2021).

[bibr6-23982128211053698] Biopac Systems, Inc (n.d.a) MB-32EEG-CAP-A ConfCap. Goleta, CA: Biopac Systems.

[bibr7-23982128211053698] Biopac Systems, Inc (n.d.b) Mobita-W-32EEG. Oldenzaal: Biopac Systems.

[bibr8-23982128211053698] Biopac Systems, Inc (n.d.c) MP160WS. Goleta, CA: Biopac Systems.

[bibr9-23982128211053698] Brain Products GmbH (2019) Brainvision Analyzer| User Manual Software Version 2.2.0. Gilching: Brain Products GmbH.

[bibr10-23982128211053698] Brain Products (2012) Brainvision Recorder & Analyzer 1.2. Gilching: Brain Products.

[bibr11-23982128211053698] Brain Products (n.d.a) Brainamp Mr Plus. Gilching: Brain Products.

[bibr12-23982128211053698] Brain Products (n.d.b) Easycap system kit. Stans: Brain Products.

[bibr13-23982128211053698] Bruininks RH Bruininks BD (2005) Bruinkinks-oseretsky Test of Motor Proficiency (2nd edn). Circle Pines, MN: NFER-Nelson.

[bibr14-23982128211053698] Charness G Gneezy U Kuhn MA (2012) Experimental methods: Between-subject and within-subject design. Journal of Economic Behavior & Organization 81(1): 1–8.

[bibr15-23982128211053698] de Cheveigné A Arzounian D (2018) Robust detrending, rereferencing, outlier detection, and inpainting for multichannel data. Neuroimage 172: 903–912. 29448077 10.1016/j.neuroimage.2018.01.035 PMC5915520

[bibr16-23982128211053698] Dienes Z (2014) Using Bayes to get the most out of non-significant results. Frontiers in Psychology 5: 781. 25120503 10.3389/fpsyg.2014.00781 PMC4114196

[bibr17-23982128211053698] DiStefano C Dickinson A Baker E , et al. (2019) EEG data collection in children with ASD: The role of state in data quality and spectral power. Research in Autism Spectrum Disorders 57: 132–144. 31223334 10.1016/j.rasd.2018.10.001 PMC6585985

[bibr18-23982128211053698] Doyle LMF Yarrow K Brown P (2005) Lateralization of event-related beta desynchronization in the EEG during pre-cued reaction time tasks. Clinical Neurophysiology 116(8): 1879–1888. 15979401 10.1016/j.clinph.2005.03.017

[bibr19-23982128211053698] Eriksen BA Eriksen CW (1974) Effects of noise letters upon the identification of a target letter in a nonsearch task. Perception & Psychophysics 16(1): 143–149.

[bibr20-23982128211053698] Fagerland MW Sandvik L (2009) The Wilcoxon-Mann-Whitney test under scrutiny. Statistics in Medicine 28(10): 1487–1497. 19247980 10.1002/sim.3561

[bibr21-23982128211053698] Faul F Erdfelder E Lang A-G , et al. (2007) G*Power 3: A flexible statistical power analysis program for the social, behavioral, and biomedical sciences. Behavior Research Methods 39(2): 175–191. 17695343 10.3758/bf03193146

[bibr22-23982128211053698] Ferree TC Luu P Russell GS , et al. (2001) Scalp electrode impedance, infection risk, and EEG data quality. Clinical Neurophysiology 112(3): 536–544. 11222977 10.1016/s1388-2457(00)00533-2

[bibr23-23982128211053698] Friedman D Cycowicz YM Gaeta H (2001) The novelty P3: An event-related brain potential (ERP) sign of the brain’s evaluation of novelty. Neuroscience and Biobehavioral Reviews 25(4): 355–373. 11445140 10.1016/s0149-7634(01)00019-7

[bibr24-23982128211053698] Hausknecht JP Halpert JA Di Paolo NT , et al. (2007) Retesting in selection: A meta-analysis of coaching and practice effects for tests of cognitive ability. The Journal of Applied Psychology 92(2): 373–385. 17371085 10.1037/0021-9010.92.2.373

[bibr25-23982128211053698] Hazarika J Dasgupta R (2018) Neural correlates of action video game experience in a visuospatial working memory task. Neural Computing and Applications 32(6939): 1–10.

[bibr26-23982128211053698] Hinrichs H Scholz M Baum AK , et al. (2020) Comparison between a wireless dry electrode EEG system with a conventional wired wet electrode EEG system for clinical applications. Scientific Reports 10(1): 5218. 32251333 10.1038/s41598-020-62154-0 PMC7090045

[bibr27-23982128211053698] Jochumsen M Knoche H Kjaer TW , et al. (2020) EEG headset evaluation for detection of single-trial movement intention for brain-computer interfaces. Sensors 20(10): 2804. 10.3390/s20102804 PMC7287803 32423133

[bibr28-23982128211053698] Kam JWY Griffin S Shen A , et al. (2019) Systematic comparison between a wireless EEG system with dry electrodes and a wired EEG system with wet electrodes. Neuroimage 184: 119–129. 30218769 10.1016/j.neuroimage.2018.09.012 PMC6568010

[bibr29-23982128211053698] Kemp B Värri A Rosa AC , et al. (1992) A simple format for exchange of digitized polygraphic recordings. Electroencephalography and Clinical Neurophysiology 82(5): 391–393. 1374708 10.1016/0013-4694(92)90009-7

[bibr30-23982128211053698] Klawohn J Santopetro NJ Meyer A , et al. (2020) Reduced P300 in depression: Evidence from a flanker task and impact on ERN, CRN, and Pe. Psychophysiology 57(4): e13520. 10.1111/psyp.13520 31898810

[bibr31-23982128211053698] Lakens D (2021, January 4) Sample size justification. Available at: 10.31234/osf.io/9d3yf

[bibr32-23982128211053698] Ledwidge P Foust J Ramsey A (2018) Recommendations for developing an EEG laboratory at a primarily undergraduate institution. Journal of Undergraduate Neuroscience Education: JUNE : A Publication of FUN, Faculty for Undergraduate Neuroscience 17(1): A10–A19. PMC6312138 30618494

[bibr33-23982128211053698] Leske S Dalal SS (2019) Reducing power line noise in EEG and MEG data via spectrum interpolation. Neuroimage 189: 763–776. 30639330 10.1016/j.neuroimage.2019.01.026 PMC6456018

[bibr34-23982128211053698] Mathewson KE Harrison TJL Kizuk SAD (2017) High and dry? Comparing active dry EEG electrodes to active and passive wet electrodes. Psychophysiology 54(1): 74–82. 28000254 10.1111/psyp.12536

[bibr35-23982128211053698] Meyer TJ Miller ML Metzger RL , et al. (1990) Development and validation of the Penn State Worry Questionnaire. Behaviour Research and Therapy 28(6): 487–495. 2076086 10.1016/0005-7967(90)90135-6

[bibr36-23982128211053698] Michael D Edwardes B (2001) Distribution-free dispersion tests for data with ties. Journal of Nonparametric Statistics 13(3): 311–330.

[bibr37-23982128211053698] Nakata H Sakamoto K Kakigi R (2015) Effects of task repetition on event-related potentials in somatosensory Go/No-go paradigm. Neuroscience Letters 594: 82–86. 25817359 10.1016/j.neulet.2015.03.055

[bibr38-23982128211053698] Oliveira AS Schlink BR Hairston WD , et al. (2016) Proposing metrics for benchmarking novel EEG technologies towards real-world measurements. Frontiers in Human Neuroscience 10: 188. 27242467 10.3389/fnhum.2016.00188 PMC4861738

[bibr39-23982128211053698] Olvet DM Hajcak G (2009) The stability of error-related brain activity with increasing trials. Psychophysiology 46(5): 957–961. 19558398 10.1111/j.1469-8986.2009.00848.x

[bibr40-23982128211053698] Pierce S Kadlaskar G Edmondson DA , et al. (2021) Associations between sensory processing and electrophysiological and neurochemical measures in children with ASD: An EEG-MRS study. Journal of Neurodevelopmental Disorders 13(1): 5. 33407072 10.1186/s11689-020-09351-0 PMC7788714

[bibr41-23982128211053698] Pinegger A Wriessnegger SC Faller J , et al. (2016) Evaluation of different EEG acquisition systems concerning their suitability for building a brain-computer interface: Case studies. Frontiers in Neuroscience 10: 441. 27746714 10.3389/fnins.2016.00441 PMC5043223

[bibr42-23982128211053698] Pratt N Willoughby A Swick D (2011) Effects of working memory load on visual selective attention: Behavioral and electrophysiological evidence. Frontiers in Human Neuroscience 5: 57. 21716633 10.3389/fnhum.2011.00057 PMC3115462

[bibr43-23982128211053698] Psychology Software Tools (2012) E-Prime 2.0. Pittsburgh, PA: Psychology Software Tools.

[bibr44-23982128211053698] Radüntz T (2018) Signal quality evaluation of emerging EEG devices. Frontiers in Physiology 9: 98. 29491841 10.3389/fphys.2018.00098 PMC5817086

[bibr45-23982128211053698] Raj V Hazarika J Hazra R (2020) Feature selection for attention demanding task induced EEG detection. In: Proceedings of the 2020 IEEE applied signal processing conference (ASPCON), Kolkata, India, 7–9 October, pp. 1–15. New York: IEEE.

[bibr46-23982128211053698] Riesel A Weinberg A Endrass T , et al. (2013) The ERN is the ERN is the ERN? Convergent validity of error-related brain activity across different tasks. Biological Psychology 93(3): 377–385. 23607999 10.1016/j.biopsycho.2013.04.007

[bibr47-23982128211053698] Rietdijk WJR Franken IHA Thurik AR (2014) Internal consistency of event-related potentials associated with cognitive control: N2/P3 and ERN/Pe. PLoS ONE 9(7): e102672. 10.1371/journal.pone.0102672 PMC4102542 25033272

[bibr48-23982128211053698] RStudio Team (2020) RStudio: Integrated Development for R. RStudio. Boston, MA: PBC. Available at: http://www.rstudio.com/.

[bibr49-23982128211053698] Tanner D Morgan-Short K Luck SJ (2015) How inappropriate high-pass filters can produce artifactual effects and incorrect conclusions in ERP studies of language and cognition. Psychophysiology 52(8): 997–1009. 25903295 10.1111/psyp.12437 PMC4506207

[bibr50-23982128211053698] Tanner D Norton JJS Morgan-Short K , et al. (2016) On high-pass filter artifacts (they’re real) and baseline correction (it’s a good idea) in ERP/ERMF analysis. Journal of Neuroscience Methods 266: 166–170. 26774028 10.1016/j.jneumeth.2016.01.002

[bibr51-23982128211053698] Topor M Opitz B Dean P (2021a) In search for the most optimal EEG method: A practical evaluation of a water-based electrode EEG system. Available at: https://osf.io/kubv5/10.1177/23982128211053698 PMC8554570 34722932

[bibr52-23982128211053698] Topor M Opitz B Leonard HC (2021b) Error-related cognitive control and behavioral adaptation nechanisms in the context of motor functioning and anxiety. Frontiers in Human Neuroscience 15: 615616. 33613211 10.3389/fnhum.2021.615616 PMC7892788

[bibr53-23982128211053698] van Dinteren R Arns M Jongsma MLA , et al. (2014) Combined frontal and parietal P300 amplitudes indicate compensated cognitive processing across the lifespan. Frontiers in Aging Neuroscience 6: 294. 25386141 10.3389/fnagi.2014.00294 PMC4208402

