# Supplementary material for: In search for the most optimal EEG method: A practical evaluation of a water-based electrode EEG system
Source: Brain Neurosci Adv. 2021 Oct 26;5:23982128211053698. doi: 10.1177/23982128211053698 (PMC8554570; doi:10.1177/23982128211053698)
Supplement: sj-docx-1-bna-10.1177_23982128211053698 – Supplemental material for In search for the most optimal EEG method: A practical evaluation of a water-based electrode EEG system [file sj-docx-1-bna-10.1177_23982128211053698.docx]

**Supplementary File**

**In search for the most optimal EEG method: A practical evaluation of a water-based electrode EEG system.**

Topor, M., Opitz, B., Dean, P. J. A.

1. **Checking Electrode Signal Quality**

It was not possible to check electrode impedance in the Mobita EEG system. Instead, we tried to ensure minimal noise by observing live spectral power for each electrode. If a power spike at 50Hz was evident, the researcher tried to improve the signal quality by taking out the electrode, moving the hair around the empty electrode gromet to expose the scalp and reinserting it again. This proved to be challenging for electrodes located at the back of the head where the hair is generally the thickest. For one participant, it was not possible to stabilise the electrodes and ensure that they do not get displaced due to hair movement. We used a bandage to secure the electrodes in place especially covering the reference electrodes located on the mastoids. Figure 1 presents an example for how the bandage was applied and how the 50Hz power changed as a result at the left reference electrode (channel 31). A video that illustrates live checking of the spectral power can be accessed from <https://osf.io/mjybt/>.

| **A**  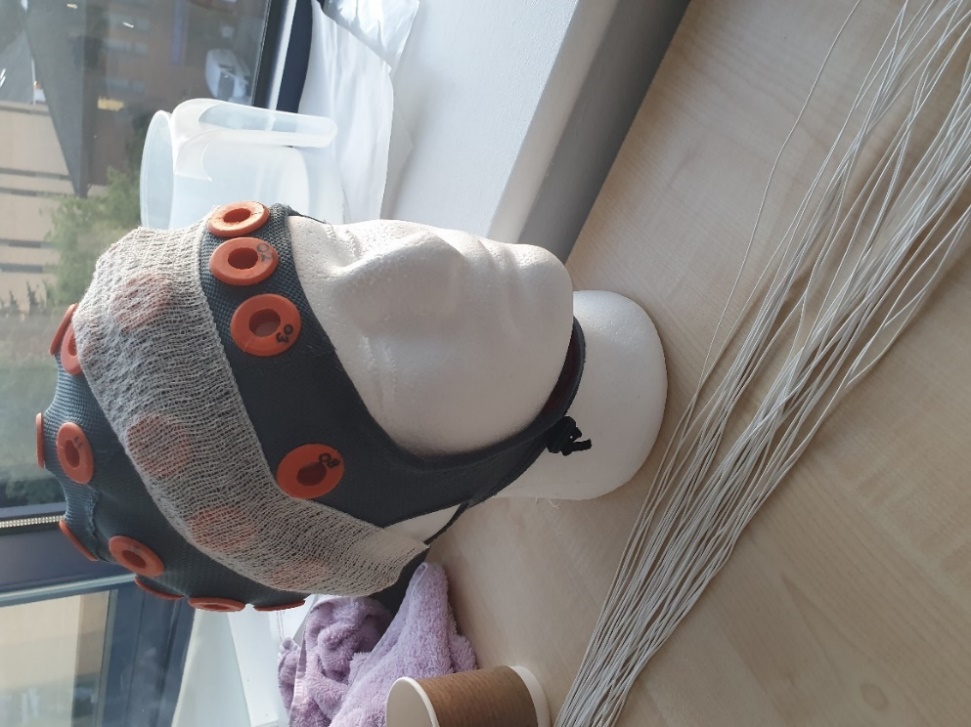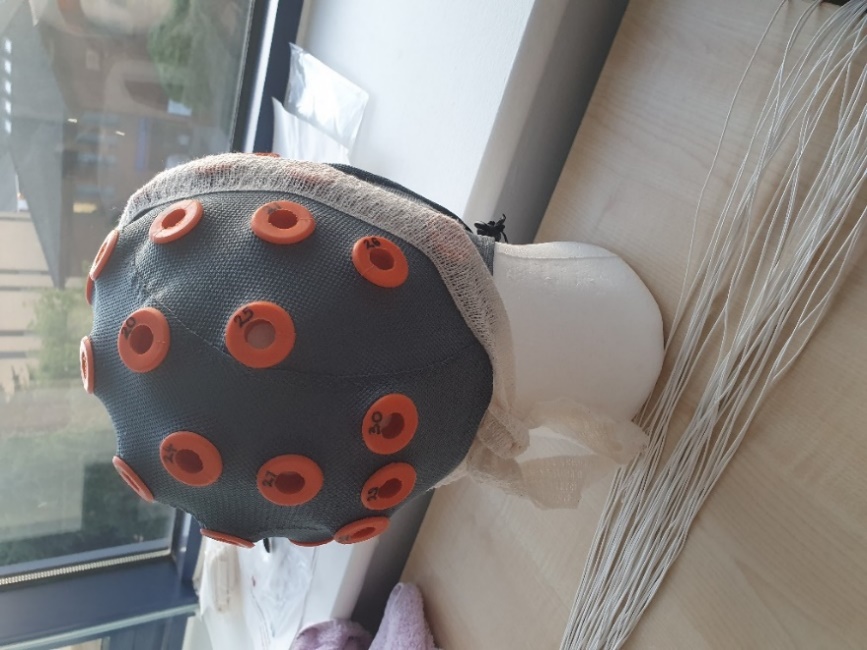  **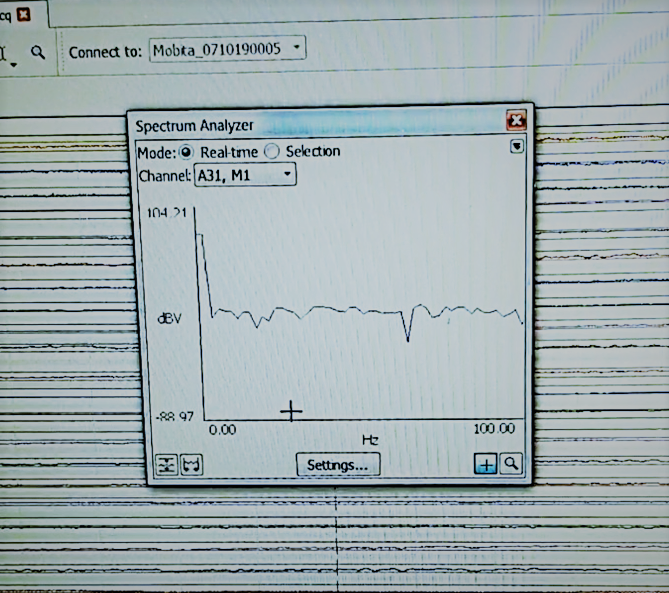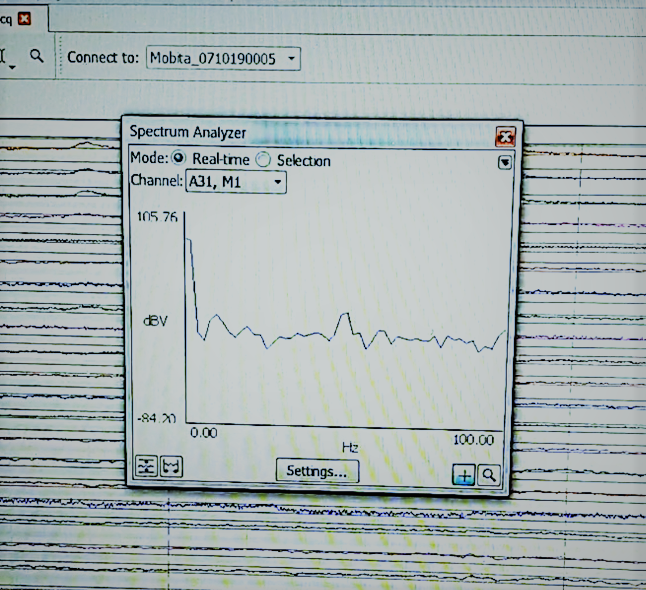B**  **Before Bandage After Bandage**  *Figure 1.* Pictures presented in **panel A** illustrate the placement of the bandage that helped to keep the reference electrodes in place throughout the recording. Pictures presented in **panel B** show the live spectral power for the left mastoid electrode (channel 31) with a small spike at 50Hz before the application of the bandage which is reduced after the application of the bandage. |
| --- |

1. **Alternative recording setup for digital event markers**

The parallel port from the stimulus presentation computer was connected to the STP100C module of the MP160 Biopac device. The STP100C was then connected to Mobita via its Solid State Relay Drive (SSRD) connection, pin 1 (SSRD1), using a long BNC cable with two touchproof leads attached to the ground and trigger sockets on the Mobita amplifier. To ensure that the SSRD1 pin gets the correct signal, the MP160 had a specially made parallel port breakout board attached with pin 3 shorted to 37 and pin 21 shorted to 33. This meant that the Mobita amplifier, that was strapped to the participant’s arm, was directly wired to the stimulus computer and the setup was no longer wireless. In a standard wired EEG system, the wired connection is between the EEG electrodes (i.e. the participant), the amplifier and the recording computer. In this specific situation, the wiring was between the stimulus presentation computer, the amplifier and the EEG electrodes (participant). Our alternative method was safe for participants as the STP100C is an isolated digital interface thus preventing the electrical current from travelling directly between the stimulus presenting computer and the Mobita electrodes.

The pictures of the breakout board are displayed in Figure 2 below. The link between pin 21 and 33 pulls digital channel 4 (D4) low, enabling the use of the SSRD connections, and the link between pin 3 and 37 connects D0 (linked to SSR1) to D8 (linked to the lowest bit on the parallel port). This sets up the MP160 so that any input received to the lowest bit on the parallel port will be routed to SSR1 and on to the Mobita. The lowest bit on the parallel port corresponds to the value 1, which means it will be active for every odd number event marker sent. This setup works, with some compromises. The recording template for Mobita only allows to enable one digital channel which means that all digital signals are either assigned the value of 0 or 1. It is therefore challenging to decode different types of events (stimuli, responses etc.). The signal appears as rectangular wave variations in the digital channel below the EEG data (see Figure 3). In the current study, we programmed the digital signal transfer so that all stimuli presented on the screen changed the value from 0 to 1 and participant responses from 1 to 0 to distinguish between the two events.

| 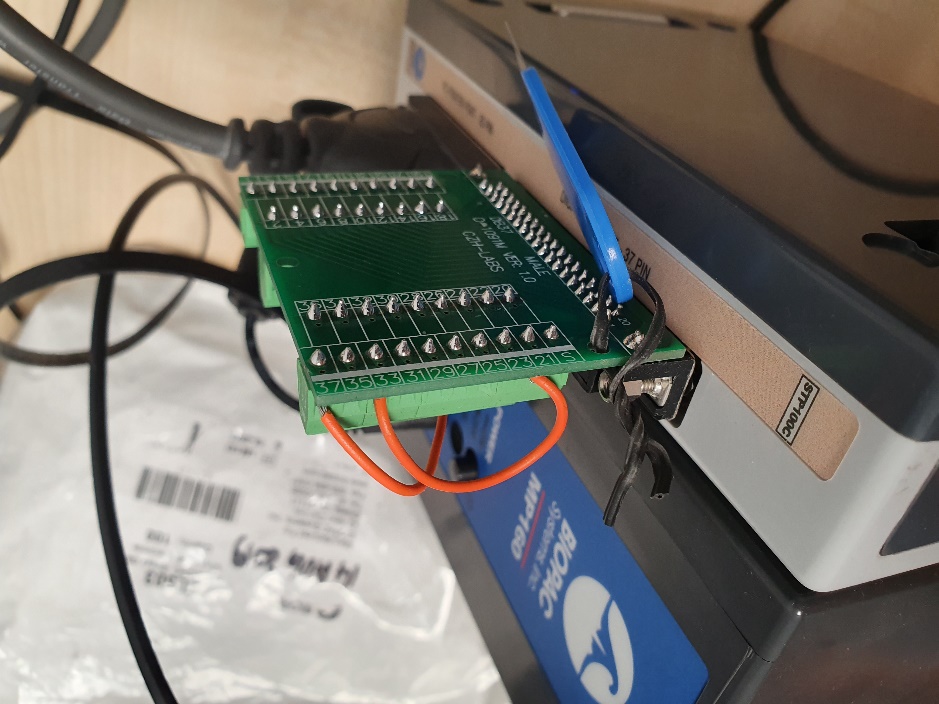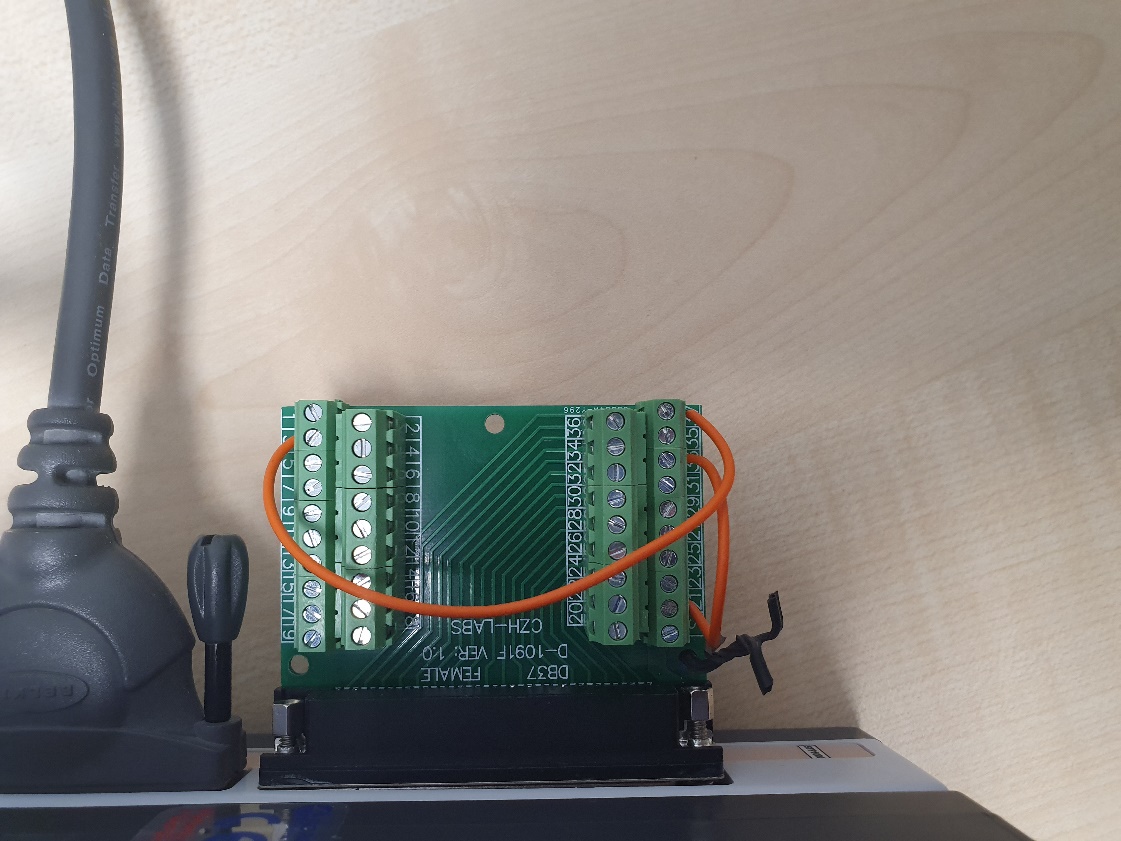  *Figure 2.* The breakout board used to establish a wired connection for the digital signal so that it could be recorded by the Mobita amplifier. The orange wires are the short-circuited connections between pins 3 and 37 and pin 21 and 33. The board is fitted at the back of the MP160 just above the parallel port socket. |
| --- |

| 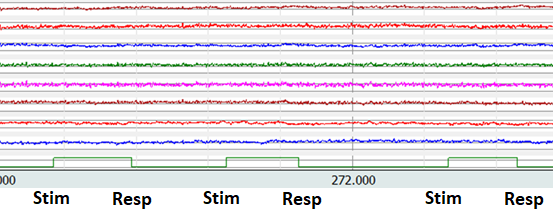  *Figure 3.* The digital event marker signal is recorded on the bottom channel in the form of rectangular shapes. We marked stimulus onset where the signal changes from 0 to 1 and response onset where the signal changes from 1 to 0. |
| --- |

1. **Digital Markers Labelling**

As evident from Figure 3 above, the digital signal is recorded alongside the EEG activity. It is not automatically turned into event markers. We followed the Acqknowledge instructions to add event markers based on the digital signal recorded. We then copied all marker information to the clipboard and exported to a .csv file. This included the marker number, onset time, type, channel, label, and date created.

All EEG data was processed and analysed in BrainVision Analyzer and the marker information that was extracted from Acqknowledge 1) did not include exact marker labels to reflect different types of stimuli (based on the task condition) or responses (correct or error) and 2) was not compatible and with BrainVision Analyzer.

To assign marker labels, the .csv file exported from Acqknowledge was combined with a .csv file exported from Eprime using a script written in R Studio. The information recorded by Eprime allowed for the identification of each marker type that occurred during the recording. Lastly, the marker file was adjusted to suit the format that was required by BrainVision Analyzer. The resulting marker files can be accessed at <https://osf.io/3wqut/> alongside the R script and the Acqknowldge and Eprime files that were used.

One thing to consider is that the synchronisation of stimuli and assigning the correct labels to markers recorded in Acqknowledge became challenging when the signal dropped during the recording. We had to manually trace the gaps and ensure that they were correctly accounted for as Acqknowledge does not record the latency or duration of signal loss or recording termination. This manual adaptation process is reflected in the R script used to create the final marker files <https://osf.io/fwbva/>.
